# Defining ‘free zones’: A systematic review of literature

**DOI:** 10.1016/j.heliyon.2023.e15344

**Published:** 2023-04-11

**Authors:** O.S. Alansary, Tareq Al-Ansari

**Affiliations:** College of Science & Engineering, Hamad Bin Khalifa University, Qatar

**Keywords:** Free zones, Free zone terminology, Free zone components, Special economic zones, Export processing zones, Free trade zones

## Abstract

For decades, the notion of ‘*Free Zones*’ has remained complex and ambiguous. Despite considerable efforts in this regard, the concept of ‘*Free Zones*’ and its components have not been sufficiently explored. To further consolidate the related literature, this study conducted a literature review on the terminology of the *Free Zone* and its components using a systematic review method based on literature and international organizations such as the UNCTAD. This study provides needed general knowledge about the nature of *Free Zones*. It also presents the classifications and types of economic zones, and provides insight into what distinguishes *Free Zones* and economic zones from one another. Furthermore, it aggregates the *Free Zone* into its constituting components, concluding with insight into future research by identifying relevant challenges and gaps.

## 1 Introduction

Recently, special economic zones have witnessed increasing importance worldwide. In line with the expansion of globalization, their number reached nearly 5,400 zones worldwide in 2018, 42.6% of which are *Free Zones* [1]. This economic activity mainly aims to achieve several goals, including attracting investments, enhancing industry, contributing to GDP, transferring knowledge, and offering jobs. However, they face many modern challenges, such as implementing governance, adapting to the new industrial revolution, and achieving sustainable development.

The maritime industry accounts for about 90% of the global trade volume as one of the important industrial sectors [2]. This type of industry includes particular categorical activities such as shipping, logistics, and supply chains [3]. Maritime transport has considerable importance in logistics and supply [4]. In this regard, the *Free Zone* is a crucial part of the maritime supply chain. According to UNCTAD [5], shipping accounts for more than 80% of international trade transport. The volume of maritime trade exceeded 10.7 billion tons in 2017, with a growth rate of 4% [6]. In line with this rapid growth and fulfillment of its requirements, the infrastructure of *Free Zones* has been developed [7]. In addition, this growth might be associated with other modern-day challenges, such as adapting to the new industrial revolution, achieving sustainable development [1], and meeting growing environmental challenges such as greenhouse gas emissions and air pollution [8,9].

Despite such significant importance of *Free Zones*, there is still ambiguity in defining the term *Free Zones* and distinguishing it from other economic zones. In this context, the study provides an overview of the *Free Zone's* definition, types, and classifications. Furthermore, it aggregates the components of *Free Zones* and examines their interaction, facilitating the improvement of performance assessment, especially with respect to sustainability, and providing a clear related understanding for academics, professionals, and decision-makers towards enhancing knowledge and promoting economic growth and development.

**Fig. 1:**
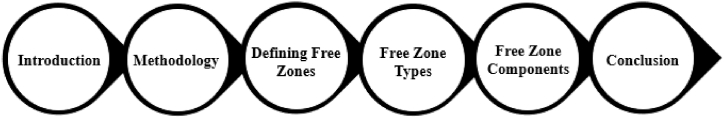
Literature review map.

To the best of the authors' knowledge, this study is distinguished from its predecessors as it provides a review of *Free Zone* definitions based on the official classification of UNCTAD in 2019. The study has two main objectives: first, providing an overview of the *Free Zones*' definitions, classifications, and types; second, aggregating components for *Free Zones*. Consequently, the first part of the review presented the terminologies of *Free Zones*, focusing on *Free Zones* and their types. Subsequently, in the second part, *Free Zones* were aggregated into their constituting components based on the UNCTAD definition in 2019 [1] and Bost's model in 2010 [10]. The structure of the review is illustrated in Fig. 1.

## 2 Methodology

To appropriately conduct a related review, a systematic literature review was performed. The section below provides more details about the methodology used. The methodology of this study consists of two parts: *Free Zone's* terminologies and *Free Zone's* components. Given the scarcity of relevant resources in the first part, the review relied on official reports from relevant organizations in addition to available reviews of literature. In the second part, the study used a systematic review to aggregate components for *Free Zones*. Initially, the research relied on both related frameworks: the UNCTAD generic framework for Special Economic Zones in 2019 [1] and Bost's model for *Free Zones* in 2010 [10]. Depending on the terms defined by the previous frameworks, the methodology used the following keyword: Export Processing Zones and Free Trade Zones. Accordingly, a search was conducted in the Scopus database for the time frame between 2010 and 2021 in the research, titles, abstracts, and keywords as follows: (export AND processing AND zones AND assessment), (export AND processing AND zones AND assessment AND literature AND review), (free AND trade AND zones AND assessment AND literature AND review), (free AND trade AND zones AND assessment).

**Fig. 2:**
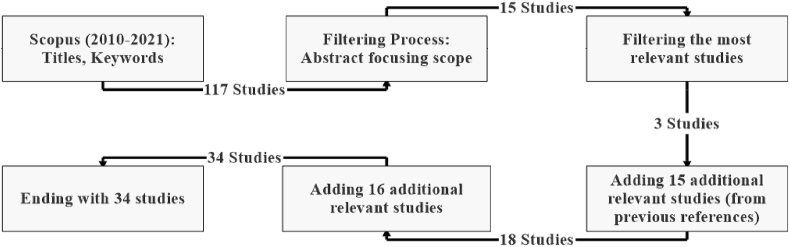
Methodology.

**Fig. 3:**
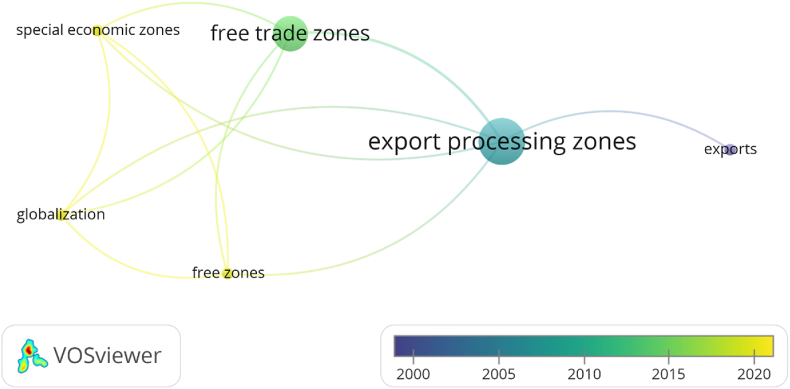
Bibliometric analysis.

Following the previous steps, the review went through six filtering stages, as shown in Fig. 2. In the first stage, 117 research studies were obtained using the above-mentioned keywords in the Scopus database. After completing the filtering process (title and abstract) in the second stage, 15 articles were selected as the most relevant. In the third stage, the articles selected in the second stage were filtered into three studies. After that, 15 additional articles were collected from the most relevant references. Similarly, 16 additional recent articles were added in the fifth stage. Finally, the methodology concluded with 34 studies. After gathering the sources, the study aggregated the components of *Free Zones* by rearranging the most mentioned components in the related literature accordingly. Fig. 3 shows the bibliographic analysis related to the literature review.

## 3 Defining ‘free zones’

This section discusses the *Free Zone's* concept, types, and components. First, it presents a historical narrative for the development of *Free Zone* terminologies. Then it details how the generations of economic zones have been developed over time, starting from the first generation prior to 1,500 AD to the sixth generation that appeared post-1990s, in addition to the period in which *Free Zones* flourished. Besides, the study statistically illustrates the global growth of special economic zones, including *Free Zones*. Subsequently, a review of the definitions and types of *Free Zones* was presented.

The ambiguity of *Free Zone*s terminology has led to many variations that have long been a subject of controversy in academic and professional circles. Hence the need for a clear definition of the *Free Zones* terminology. In this context, the paper presents a historical narrative of the concept of *Free Zones* until 2019, when the official classification was proposed by UNCTAD, whereby a range-wide of special economic zones were defined, including *Free Zones*. Moreover, components for *Free Zones* were aggregated based on UNCTAD's generic classification in 2019 [1] and Bost's model in 2010 [10].

### 3.1 Free zone terminology

The concept of *Free Zones* dates back more than 2,000 years [11], when goods were transported by ships for importing and exporting purposes with little or no intervention from local authorities [1]. Although the *Free Zones* term is relatively old, its use in modern international trade is considered a recent invention [12]. The *Free Zone* terminology likely originated from areas located on international trade routes such as Longhorn (1547), Marseilles (1669), Gibraltar (1704), Singapore (1819), Hong Kong (1848), Hamburg (1888), and Copenhagen (1891). In 1959, Ireland was the first county to establish a modern *Free Zone* [13]. According to UNCTAD [1], the 1960s witnessed the beginning of the emergence of the modern form of *Free Zones* as locations close to airports, seaports, and border corridors.

The term ‘freedom’ or ‘free’ is strictly limited. Usually, it does not extend beyond the formalities specified by customs regulations [12]. ‘Freedom’ is defined as the financial incentives and special administrative and economic privileges governments provide exclusively to *Free Zones,* but not other zones in the same local economies [14]. Moreover, the national tax system and the other relevant economic and administrative restrictions do not apply to these zones. This kind of freedom is supposed to assure an unrestricted flow between global zones and economies of capital, people, goods, services, and other administrative privileges [15]. As for some other relevant laws like public health, passenger rights, and ship entry permits, they are subject to the directives of the local authorities. Thus, it can be inferred from such a definition of freedom that *Free Zones* enjoy limited freedom within the rights framework of host countries.

**Fig. 4:**
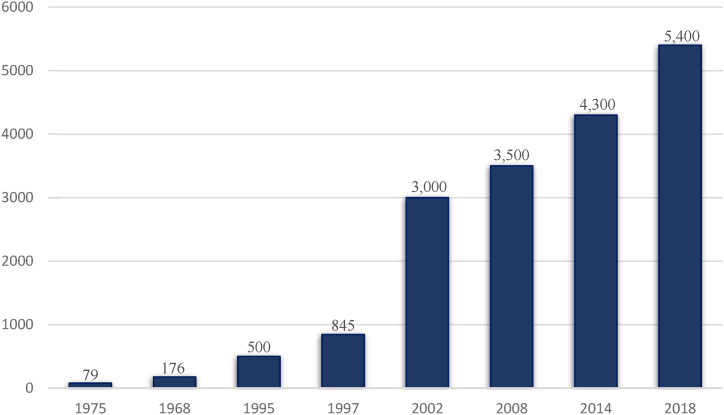
Historical trend of SEZs worldwide [1].

As illustrated in Table 1, the concept of a *Free Zone* has developed over time and taken many different forms of zones such as free trade zones, bonded warehouses, free ports, export processing zones, and broader economic development zones [16,17]. However, the general concept underlying all zones is to offer certain privileges to the participating companies on the condition that they engage in any form of international activities [18]. By offering such privileges, host countries expect to obtain other benefits from the *Free Zones* towards developing their domestic economic growth, such as increasing competitiveness development in the export market [[19], [20], [21]], achieving economic diversity through introducing new activities and sectors into the country [10,22,23], increasing foreign exchange earnings, providing job opportunities, attracting technology, and knowledge transfer. In 1935, the total number of economic zones was restricted to 51 [12]. However, the 1990s witnessed a massive increasein special economic zones, specifically between 1997 and 2002, when their number rose from 845 to 3,000 zones. Such a trend continued to grow in many countries reaching approximately 5,400 SEZs in 2018, 42.6% of which were *Free Zones,* as illustrated in Fig. 4.

### 3.2 Free zone generations

This historical development of economic zones, as outlined in Table 1, spans several centuries, beginning with the appearance of primary economic zones in the form of free cities and free ports before the 1550s. Over time, economic zones have evolved and expanded in scope and purpose, reflecting economic and technological changes and seeking to enhance international economic integrations for host countries.

**Table 1:** SEZ's Generations. Source (UNCTC, 1990; Kreye et al., 1987; Chen, 1995 [24]; Wong & Chu, 1984 [25]; Yuanyang et al., 1993): as cited from Meng [15].

FEZ’ Generations	Primary FEZs	(1st) Generation Trade-based FEZ	(2nd) Generation Manufacture-Based FEZ	(3rd) Generation Service-focused FEZ	(4th) Generation Science-focused FEZ	(5th) Generation Comprehensive FEZ	(6th) Generation Cross-border FEZ
Period	Pre 1500s	Pre 1950s	Post 1950s		Post 1980s		Post 1990s
	Free cities. Free ports.	Freeports.	Duty-free export processing zone. Export Free Zone. Export processing zone. Free export processing zone. Free export zone. Free production zone. Industrial exporting processing zone. Industrial Free Zone. Investment promotion zone. Joint enterprise zone. Free enterprise zone. Zone of joint entrepreneurship. Privileged export zone. Maquiladoras import processing zone. Agricultural export processing zone. Free agricultural zone. Economic & technological development zone.	Free professional zone. Free service zone. Free banking zone. Free insurance zone. Free red-light zone. Free gambling zone. Free medical zone. Free tourist zone.	Incubator. Research Park/areas. Research triangle. Technology parks. Science Park/Scientific parks. High-tech parks. Science-based parks. Science & technology park. Science-based industrial park. Tech-development parks. New and high technologic development zone. Science city/Science town. Technopoleis. Academic town. Silicon Village/Silicon Island. High-tech industrial area.	*Free Zone*. Free economic zone. Special economic zone. Comprehensive free port. Comprehensive free trade zone.	Growth triangle. Cross border economic cooperation zone. Cross national regional economic integration. World economic integration.
		Free city/state.					
		Bound house zone.					
		Customs bonded warehouse.					
		Customs Free Zone.					
		Customs zone.					
		Duty-free zone.					
		Tax-free trade zone.					
		Tax-free zone.					
		Free trade zone.					
		Foreign trade zone.					
		Free border zone.					
		Transit Zone.					
		Transshipment zones.					
		Free frontier zone.					

The first generation of economic zones, which emerged before the 1950s, was limited to trade-based activities such as freeports and customs-bonded warehouses. These zones were established to facilitate international trade and reduce the cost of importing and exporting goods. After the 1950s, the second and third generations of economic zones emerged, focusing on manufacturing and services, respectively. This shift was driven by the desire of many countries to promote industrialization and service-based economies [26] to enhance the development of various services sectors, such as banking, insurance, and tourism [15]. The fourth and fifth generations appeared after the 1980s, focusing on science and comprehensive activities. Such development was driven by the rapid pace of technological advancement and the need to develop knowledge-based economies.

The fourth generation of science-focused economic zones was aimed at fostering innovation and promoting high-tech industries, while the fifth generation of comprehensive economic zones sought to provide a more comprehensive approach to economic development by providing a wide range of economic activities, including trade, manufacturing, and services [26]. In this context, *Free Zones* appeared with the aim of helping achieve such purposes [15]. Finally, the sixth generation of cross-border economic zones emerged after the 1990s. These latest generations are focused on cross-border economic cooperation and integration. They are driven by the increasing globalization of trade and commerce, aiming to promote economic growth and integration across different regions and countries. In conclusion, the historical development of economic zones reflects the changing needs and priorities of the global economy, with each generation building upon the successes and lessons of previous generations, considering renewed needs to create comprehensive and integrated growth, industrialization, and technological advancement, as well as to support transboundary economic integration and cooperation.

### 3.3 Free zones definitions

In the literature, many definitions and terms addressed the concept of *Free Zones*. Although most of these definitions were reasonably precise in describing *Free Zones* as areas facilitating the transit of goods; however, they noticeably vary in some other details, especially in services. In this section, this study presents literature related to the concept of *Free Zones* from the earliest definitions to the most recent.

Amongst the earliest attempts to define *Free Zones* was the definition introduced by the US Tariff Commission on behalf of the US Congress in 1934. In this context, the Free Zone was described as an area of limited size that is exempt from customs regulations for goods intended for re-export; with freedom from customs duties as long as the imported goods remain within the defined area - which is typically located near a port of entry and does not have a permanent population-; equipped with necessary facilities for loading and unloading, fuel and ship supplies, and storage for goods that will eventually be re-shipped by both land and water; where goods can be landed, stored, mixed, blended, repacked, manufactured, and re-shipped without paying duties and without the intervention of customs officials [12]. From this definition, it could be concluded that *Free Zones* are subject to all laws except customs. Another definition of *Free Zones* was proposed by the US Council in 1988, which described a *Free Zone* as a separate part of the customs territory of the community and is differentiated from the rest of the territory; non-community goods placed in these zones are treated as if they are not within the customs territory of the community for the purposes of determining import duties and commercial policy import measures; this is provided that these goods are not released for free circulation or entered using another customs procedure according to the regulations outlined in the governing document [27].

In his definition of *Free Zones*, Querci [11] focused on the laws' independence of *Free Zones* from other local areas of the host country. According to Querci [11], a *Free Zone* can be described as an area that is considered to be an international territory and is exempt from the jurisdiction of the state in terms of the flow of goods and services, customs regulations, labor laws, and economic laws; it operates as an autonomous legal system that is primarily governed by international trade customs and practices [11]. Similarly, McCalla focused on the regulation's perspective of *Free Zones* definition. Based on McCalla's definition, *Free Zones* are *“designated areas in which some relaxation of national laws or regulations, be they customs duties, income tax regulations, banking regulations, or minimum wage levels”* [28].

On the other side, it is noticeable that most *Free Zone* definitions agree that a *Free Zone* should be dedicated to two basic things: fenced geographical area and incentives. Corresponding to Madani [29], a *Free Zone* is described as a geographically defined and enclosed area within a country where business activities are encouraged through a set of policy measures and incentives to attain specific economic goals*.* Likewise, the Revised Kyoto Convention emphasized in its definition of *Free Zones* on the fenced geographic area and regulations. Likewise, the Revised Kyoto Convention emphasized in its definition of *Free Zones* on the fenced geographic area and regulations. As per the Revised Kyoto Convention, a *Free Zone* is “*a part of the territory of a contracting party where any goods introduced are generally regarded, insofar as import duties and taxes are concerned, as being outside the customs territory*” [30]. The European Union summed up these two main conditions for *Free Zones* by proposing a more specific *Free Zone* where it defines *Free Zones* as: *“enclosed areas within the customs territory of the Union where non-Union goods can be introduced free of import duty, other charges (i.e., taxes), and commercial policy measures”* [31].

In addition, it should be noted that *Free Zones* are mainly established to facilitate trade by allowing fewer customs formalities. Accordingly, goods placed within such areas are free of import duties, value-added tax, and other import duties [32,33]. In a similar manner, Bost offers a definition similar to earlier definitions of *Free Zones*. Referring to Bost [32], the term *Free Zone* can be defined as “*an area of variable size, in which authorized companies are exempt from the normal regime applicable in the host country, in particular with regard to customs (or even taxation where the country authorizes)*”. Besides, The World Free Zone Organization (WFZO) developed a more comprehensive definition of *Free Zones*, adding other economic activities and services. Referring to WFZO [34], *“a Free Zone is an area designated by one or more government(s) where economic activities, whether production or trade, physical or virtual with respect to goods, services or both, are permitted and relieved (totally or partially) from customs duties, taxes,* fees *or with specific regulatory requirements that would otherwise apply*”. In its report in 2008, the World Bank adopted the definition of *Free Zones* proposed by the Revised Kyoto Agreement of the World Customs Organization (WCO) as “*outside the customs territory for the assessment of import duties and taxes; Free Zones typically permits for duty- and tax-free imports of raw and intermediate materials and capital equipment*” [13]. In its official classification for *Free Zones* in 2019, UNCTAD proposed the official definition of *Free Zones*. According to UNCTAD [1], *Free Zones are* “*essentially separate customs territories, in addition to relief from duties and tariffs, most zones also offer fiscal incentives, business-friendly regulations with respect to land access, permits, and licenses, or employment rules, and administrative streamlining and facilitation*”.

As explained in this section, the literature provides multiple definitions for *Free Zones*, highlighting the various interpretations and perspectives of the concept. According to the definitions provided, there is agreement that *Free Zones* are exempt from certain regulations, laws, and duties, particularly customs regulations and duties. The earliest definition provided by the US Tariff Commission in 1943 [12] emphasized the limited size of the *Free Zone*, its location near a port of entry, and the freedom from customs duties as long as the imported goods remained within the defined area. Furthermore, the definition by the US Council in 1988 [27] emphasized that *Free Zones* are considered a separate part of the customs territory and are treated as if they are not within the customs territory in terms of determining duties. Querci [11] and McCalla [28] stressed the independence of *Free Zones* from the jurisdiction of the host country and the relaxation of national laws and regulations, respectively. Madani [29] and the Revised Kyoto Convention [30] emphasized the fenced geographical area and regulations as essential aspects of *Free Zones*. The European Union [31] and the World Free Zone Organization [34] expanded on this idea by adding that *Free Zones* are enclosed areas within the customs territory of a country or union, with relief from import duties, taxes and commercial policy measures. In addition, the World Bank [13] and UNCTAD [1] added that *Free Zones* offer fiscal incentives, business-friendly regulations, and administrative streamlining and facilitation. In conclusion, the literature provides a wide range of various definitions of *Free Zones*. The majority agree that *Free Zones* are areas exempt from specific regulations, laws, and duties to facilitate trade and support economic activities. Accordingly, a *Free Zone* can be defined as a specific geographic scope where governments support economic activities -whether commercial or industrial- through offering a series of privileges focusing mainly on exemptions from customs duties and tax incentives and extending to include a wide range of support such as facilitating administrative procedures, permits, licenses, and consultations.

### 3.4 Free zone types

In the literature, there are many classifications of *Free Zones*. This review details four kinds proposed by Hibbard [12], the United Nations [35], McCalla [28], and Bost [10]. Referring to Hibbard [12], *Free Zones* can be categorized into three types: (1) Purely transit *Free Zones*. This type of *Free Zones* aims to facilitate goods' transit where products are stored to be re-exported; (2) Transit and Industrial *Free Zone*. In this kind of *Free Zones*, goods could be subject to modifications, manufacturing, and manipulation before being exported to their new destinations; and (3) *Free Zones* with nationality restrictions. Some *Free Zones* are open to all nations, while others are limited to a certain number of countries.

In 1990, the United Nations developed a new classification of modern *Free Zones* [27]. In this proposal, *Free Zones* are grouped into three main categories: (1) Trade zones exempted from customs: these zones allow entry of materials, goods, merchandise, and goods issued abroad without customs duties with the condition of being later refined, processed, or exported. Besides, payment of duties on goods is performed only if the goods are sold in the local market; (2) New Zone Types. These types of zones differ from the classical types in terms of decision-making capabilities and the ability to do business in a wide range of industrial activities, from manufacturing to services, such as enterprise parks and science and technology parks; and (3) Other types of zones. These zones include limited areas with specific elements of the operations areas, such as special economic zones, import processing areas, and regional development areas.

**Fig. 5:**
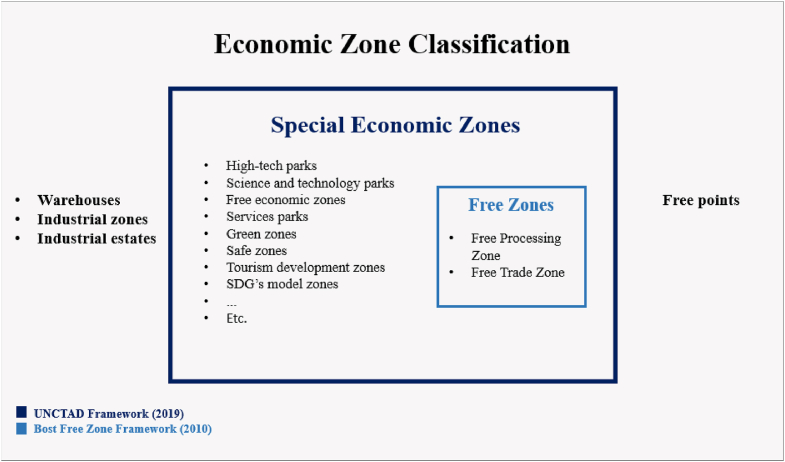
Economic Zone Classification [1]. Developed from Bost & Piantoni: Reims University [32].

Another classification of *Free Zones* was suggested by McCalla [28], who divided *Free Zones* into two main categories: (1) Associated with ports; this kind of *Free Zones* includes three types, (a) entire port areas (free ports), (b) port zones for selected activity (excluding manufacturing) contain duty-free shops, transit zones, and enterprise, (c) port zones for manufacturing including export processing zones, foreign-trade zones, and free trade zones; (2) *Free Zones* in other locations: this type of zones includes three other types of *Free Zones;* (a) remote or separated locations including free gambling zones and free perimeters, (b) Zones with cities including free enterprise zones, free banking zones, and international financial centers, (c) Nation-states including free banking zones and tax-free havens. The fourth classification was proposed by Bost [10], who suggested classifying *Free Zones* into only two main categories: Free Trade Zones and Export Processing Zones, as shown in Fig. 5. This classification will be explained in detail in the upcoming sections.

### 3.5 Types of economic zones

There are many types of economic zones in literature. This section considers a set of economic zone types mentioned by the World Bank [13], DAPhNE [33], and UNCTAD [1]. Under the generic term ‘special economic zone’, the World Bank (2008) classified economic zones into seven basic types: (1) Freeports, (2) Free Trade Zones or Commercial Zones, (3) Export Processing Zones, (4) Enterprise Zones, (5) Single Factory Exporting Processing Zones, (6) Hybrid Logistic, and (7) Special Economic Zones.

#### 3.5.1 Freeports

Freeports usually include large areas. They typically encompass the broadest possible activities, including tourism and retail sales, and wider scope of benefits and incentives [13]. In the Foreign Trade Zone report made by the US War Department and the US Shipping Board in 1929, Freeport was defined as *“a segregated area in which subject to varying restrictions as to sorting, grading, re-parking, manipulation, and manufacture, and in which such goods or authorized manufactures”* [12]. Consequently, customs procedures and applicable fees may be imposed on similar goods entering the customs territory. The freeport concept is considered older than a *Free Zone*. According to Hibbard [12], the main difference is the area assigned to each other; while the freeport includes an entire municipality, the *Free Zone* is limited to ports’ facilities.

#### 3.5.2 Free trade zones

Free Trade Zones (FTZs) are also known as Free Commercial Zones or Commercial-free Zones. Most Free Trade Zones flourish in developing countries and mainly focus on import and export activities [36]. FTZs aim to facilitate importing of foreign goods by eliminating normal customs restrictions [37,38]. Referring to Chiu et al. [36], free trade zones are a kind of special zones that aim to attract businesses and foreign investments by offering privileges such as reducing bureaucratic restrictions and eliminating regular customs tariffs and quotas. The World Bank defined FTZ as *“small, fenced-in, duty-free areas, offering warehousing, storage, and distribution facilities for trade, transshipment, and re-export operations, located in most ports of entry around the world”* [13]. By the nature of their activities, such as transshipment, re-export, and international trade, Free Trade Zones can be considered global trade hubs. Free Trade Zone plays a vital role as a facilitator for trade-in globalization [1]. The main objective of FTZs is to support trade activities focused on local and foreign markets. Usually, the typical locations for Free Trade Zones FTZs are inside or near seaports, airports, and border zones or along the main transportation axes (maritime, rail, and road) and development corridors [32].

#### 3.5.3 Export processing zones or (industrial estates)

Export processing zones (EPZs) or so-called industrial estates are “*fenced-in industrial estates specializing in manufacturing for export and offering their resident firms free-trade conditions and a liberal regulatory environment”* [39]. The activities of Eexport Processing Zones primarily focused on foreign markets [1]. Such zones seek to encourage manufacturing and related activities by providing incentives and facilities primarily aimed at export markets [13]. Geographically, Export Processing Zones can be located anywhere [32]. Besides, the World Bank [13] suggested a typical size for the Export Processing Zones with less than 100 ha. In general, Export Processing Zones have two types: the traditional EPZs, and Hybrid EPZs.

##### 3.5.3.1 Traditional EPZs

In the traditional EPZ type, the whole area inside the zone is “*exclusively for export-oriented factories licensed under an EPZ regime*” [13].

##### 3.5.3.2 Hybrid EPZs

Hybrid EPZs are divided into two main zones. The first zone is a general EPZ area open to all types of industries. The second is a separate EPZ area specialized only for export-oriented, EPZ-registered enterprises [13].

#### 3.5.4 Enterprise zones

The purpose of establishing Enterprise Zones was to revitalize distressed urban or rural areas by providing a combination of privileged forms of financial grants and tax incentives [13,33]. Such zones are commonly widespread in developed countries such as the UK, France, and the USA.

#### 3.5.5 Single factory EPZ schemes

These kinds of zones provide a set of incentives to individual factories regardless of their location. Such enterprises do not have to be located within a designated zone to enjoy the incentives and privileges [13]. Good examples are Mauritius, Madagascar, and Mexico.

#### 3.5.6 Hybrid logistic zone

A Hybrid Logistic zone was recently proposed in 2018 by Danube Transnational Program in the European Union (DAPhNE) to increase the attractiveness of ports. According to DAPhNE [33], a hybrid logistic zone is a designated area, typically surrounded by a fence, where a cluster of activities and companies involved in manufacturing, trade (primarily export), freight distribution, transportation, logistics, and related support services is encouraged through the provision of free-trade conditions, a permissive regulatory environment, and various fiscal and financial incentives.

#### 3.5.7 Special economic zones (SEZs)

Special Economic Zones, or what the World Bank calls ‘Special Zones', have existed for over one hundred years [33]. A Special Economic Zone is a type of economic zones that includes a large variety of activities, such as science & tech parks, logistics parks, airport zones, petrochemical, and smart zones [13]. In addition, SEZs are considered an effective governmental tool for attracting investments, which boosts industrial production and economic growth [19,23,40]. According to the World Bank [13], the first modern zone appeared in 1959 in Ireland. Since then, many other zones have emerged under the special economic zones concept. The prominent growth in international production in the 1990s and 2000s has led to a massive increase in the spread of special economic zones around the globe and particularly in developing countries. The latter have leveraged this opportunity to take advantage of the SEZs integration, which can provide an innovation-based multi-industry environment [1]. In the last five years, more than 1,400 SEZs have been established, representing more than 25% of the total 5,400 SEZs in 147 countries in 2019 [1]. Essentially, SEZs provide a variety of incentives, including fiscal, regulatory, and infrastructure support [32].

Special economic zones can be defined as *“a geographic area within the territory of a country where economic activities of certain kinds are promoted by a set of policy instruments that are not generally applicable to the rest of the country”* [41]. According to the United Nations Conference on Trade and Development (UNCTAD), Special Economic Zones are *“geographically delimited areas within which governments facilitate industrial activity through fiscal and regulatory incentives and infrastructure* support*”* [1]*.* Special Economic Zone (SEZ) is a generic term [13,32] that includes the recent developments of traditional zones such as free trade zones, export processing zones, industrial parks, and *Free Zones* [1,32]. The most popular type of SEZs is a *Free Zone*, which has separate customs areas [32]. Moreover, all special economic zones derived their concept from the *Free Zone*: free from taxes, tariffs, and red tape and shared the basic components of incentives [1]. SEZs have specific principles, including the following: (1) Specific geographical area, usually physically secured ‘fenced-in’; (2) Single administration (3); Eligibility of benefits based on the physical location within the zone; and (4) Isolated customs area (duty-free benefits) and streamlined procedures [13]. Many recent SEZ types focus on new industries such as high-tech, financial services, or tourism. Others focus on environmental performance and science commercialization.

Referring to UNCTAD [1], special economic zones are classified based on the following. (1) Objectives of industrial focus, such as high-tech parks and services parks, (2) Locations, such as port-based zones and border zones, (3) Types of regulatory regimes, such as Free Trade Zones. Although there are several types of Special Economic Zones, *Free Zone* is the most popular subtype of the SEZs [1]. SEZ types have been developed to meet the needs of other industries and activities [1]. For example, High Technology or Science Parks were proposed to meet the needs of science-based industries. Furthermore, the Financial Services Zone was suggested to support offshore financial and non-financial activities. Software and Internet Zone was also established to meet the needs of software coding and other offshore ICT services operations. Besides, Logistics Parks or Cargo Villages were developed to support supply chain and logistics activities. The same principle applies to SEZ types such as petrochemical, airport-based, tourism zones, SDGs model zones, etc. In 2019, UNCTAD proposed SDG model zones. This new model of special economic zones relies on innovation in their strategic focus, design, management, and operations [1]. This zone focused on three main components: (1) Focusing strategically on encouraging investments related to sustainable development goals; (2) Raising the level of environmental, social, and corporate governance standards and compliance; and (3) Enhancing comprehensive growth via relations and spillover.

## 4.0 The need for a clear definition of free zones

There have been various attempts to define *Free Zones* in academic literature. Most such efforts were developed by academics and related international organizations [42]. Despite these efforts, *Free Zones'* underlying concept remained unclear to professionals and academics [11,15]. The core dilemma occurs in the difficulty of understanding these terms, distinguishing between them, and knowing their pros and cons to evaluate their feasibility in host nations. According to Borozan & Klepo [27], the complexity of the *Free Zones* terminology is due to their historical development. Although most of its definitions are reasonably precise in describing the *Free Zones* terminology as areas facilitating the transit of goods, they widely differ in other details, especially in services [12]. Referring to Bost [32], there are three reasons behind the confusion of *Free Zones* terminology or the ‘terminological anarchy’: (1) The lack of knowledge of modernly used terminologies; (2) The tendency to propose different terms accurately reflects local capabilities; (3) Desires to present the zones more modernly from a marketing perspective, such as advantaging from using the ‘technology parks’ term and avoiding the negative reputation associated with *Free Zones* during the 1970s and 1980s, especially in terms of various labor rights.

Despite this variation in the conceptual understanding of the zone, the difference between the *Free Zone* and Special Economic Zones terms has not been addressed academically [32]. Fortunately, in 2019, UNCTAD proposed the general term ‘special economic zones’ (SEZs) in its annual report ‘WIN2019’ as an umbrella term for various economic zone types. Since then, many publications have begun to adopt the term Special Economic Zones as a more comprehensive concept that includes the *Free Zone* [32]. This new term ‘SEZ’ aims to be more inclusive than the term *Free Zones,* which no longer reflects the wide range of new zones, specifically those oriented towards technologies and services such as tourism, security, and sustainable development goals. In addition to *Free Zones*, SEZs include a wide range of zones, such as free economic zones, tourism development zones, science parks, science & technology zones, high-tech parks, safe zones, and green zones, etc. [1]. Referring to Bost [32], the use of SEZs term facilitates distinguishing between the new zones focusing on science, technology, and advanced services areas, regular industrial parks, and the other industrial zones located on the outskirts of countries with no special regulatory frameworks except zoning laws. For this reason, they cannot be considered special economic zones. The term SEZs includes *Free Zones* as a sub-type within its framework, considering it a separate customs zone [1]. *Free Zones* offer low or no customs duties for goods manufactured, assembled, or even in transit, which is the main difference between *Free Zones* and other types of SEZs.

### 4.1 UNCTAD-2019 classification

As per the definition issued by UNCTAD [1], the SEZ is the largest zone group. The core interest of SEZ is mainly focused on *Free Zones* (its largest sub-type) in addition to services and advanced technologies. Fig. 5 illustrates the new categorization of zones redesigned by Bost [32]. Generally, economic zones are classified into five major types of zones: (1) special economic zones, (2) warehouses, (3) industrial zones, (4) industrial estates, and (5) free points.

The largest group of zones is the Special Economic Zone group, which includes all the zones mentioned in UNCTAD's related definition of SEZs as *“geographically delimited areas within which governments facilitate industrial activity through fiscal and regulatory incentives and infrastructure* support*”.* SEZs include the sub-set of *Free Zones* and other special zones that benefit from incentives and are focused on advanced technologies. The *Free Zone* subgroup contains three types: Export Processing Zones, Free Trade Zones, and single factory *Free Zones*. All such types of *Free Zones* follow the UNCTAD's definition of a Free Zone as *“essentially separate customs territories, in addition to relief from duties and tariffs, most zones also offer fiscal incentives, business-friendly regulations regarding land access, permits, and licenses, or employment rules, and administrative streamlining and facilitation”* [1].

Usually, ‘Single-enterprise Free Points' are private and insignificant in terms of their contribution to countries' GDP due to their small sizes compared with multi-enterprise *Free Zones* that may include hundreds. This exception in *Free Zones* may encourage countries not to allow Single-enterprise to establish themselves in the form of ‘free points’ [32]. The single-enterprise free-point provides SEZ incentives to individual enterprises regardless of location [1,13]. According to UNCTAD [1], warehouses are a separate group. Although warehouses are not integrated into *Free Zones* in many cases, such as (alcohol and cigarettes), they are neither included under the SEZ group nor the *Free Zone* subgroup, even though they enjoy some temporary tax on goods in transit. Switzerland had 240 warehouses in 2019 with the same categorization [32]. The different and not well-known inventory procedures in many countries make it complicated to have a general understanding of the inventory of *Free Zones* [32]. The same principle is applied to the Single-enterprise Free Point, which provides SEZ incentives to individual enterprises regardless of location [1]. Free points do not refer to a specific location or area; however, it refers to a legal status given to firms to be free to set up wherever they like within the country's territory, such as being close to the raw materials or in the less attractive cities [32]. Companies that choose to be in this option have the same legal situation (benefits and constraints) as those in the *Free Zones* [10]. Unlike the single-factory free zone, UNCTAD has not officially recognized free points as zones even though they share a similar situation. According to Bost [32], this is the reason behind classifying the free points within the same category as SEZs because of the customs advantage characteristics. Although some counties offer only Free Point options, others offer both options (free zones and free points) to be more attractive for investments [32]. In the UNCTAD classification, Free Points are not considered *Free Zones*, although they are similar in incentives and restrictions [1].

In line with the basic definition of *Free Zones* agreed upon by much of the literature as separate customs zones that benefit from the advantages of customs import. Bost [32] proposed a framework for *Free Zones* that includes two main categories: Free Trade Zones and Export Processing Zones, as illustrated in Fig. 5. Although related institutions and organizations have attempted to define the concept of *Free Zones* for a long time, it remained unclear to many academics and professionals. Recently, in its annual report ‘WIN2019’, UNCTAD proposed a comprehensive framework for economic zones that generally defined and explained the differences between zones, including *Free Zones*. However, despite the UNCTAD framework offered to clear the confusion concerning the *Free Zones* concept, there is still a need for more efforts to define the components of various zone types in general and the *Free Zones* in particular. Thus, there is a need for further elaboration on the components of zones/Free Zones. Such efforts could help to provide a deeper understanding of the *Free Zone* components and facilitate the evaluation process.

### 4.2 Free zone components matrix

After a long period of discussion related to definining *Free Zones*, in 2019, UNCTAD proposed a comprehensive framework for economic zones, where the types of zones were clearly defined, including *Free Zones*. Although such a framework contributed significantly to providing clarity on the *Free Zones* concept and distinguishing it from other types of zones more precisely, the framework can continue to be advanced considering the components of *Free Zones*, which can contribute to facilitating assessment and other purposes. This section provides further insight into the components of the *Free Zones*, which is unique, specifically after the new official classification of UNCTAD in 2019.

To further suggest components for *Free Zone*, the methodology used by the review relied on the UNCTAD General Framework for Special Economic Zones in 2019 [1] and Bost's related model in 2010 [10]. In 2019, UNCTAD set a general framework for zones. This framework identified the *Free Zone* framework through three zones: Free Trade Zones, Export Processing Zones, and Single Factory Free Zone. However, the methodology adopted Bost's proposal for *Free Zone* by including the two main components mentioned in Bost's model for *Free Zone*: Export Processing Zones and Free Trade Zones and excluding the third component, as illustrated in Fig. 5. Accordingly, a search in the Scopus database was conducted during the time frame between 2010 and 2021 (see section 2). After gathering the sources, the components of the *Free Zone* were *aggregated* by rearranging the most mentioned components in the related definitions within the literature. This section lists these definitions, and then the extracted component matrix, as illustrated in Table 2.

**Table 2:** Free zone components matrix.

Components Authors	Regulations & Incentives		Trading facilities			Processing facilities				
	Fenced-in area	Regulations & Incentives, such as rules, taxes, tariffs, and services.	Warehouses	Distribution	transshipment	Packaging	Assembling	Manufacturing facilities	Repairing	Labeling
[12]	✓	✓	✓	✓	✓	✓	✓	✓	✓	✓
(UNCTC, 1988 via [18])	✓	✓	✓	✓	✓	✓	✓	✓	✓	✓
(Querci, 1989 via [11])	✓	✓								
[43]	✓	✓	✓		✓	✓	✓	✓	✓	✓
[28]	✓	✓								
[39]	✓	✓	✓	✓	✓	✓	✓	✓	✓	✓
[44]			✓	✓	✓	✓	✓	✓	✓	✓
[45]	✓	✓	✓	✓	✓	✓	✓	✓	✓	✓
(ILO, 1998 via [11]).	✓	✓	✓	✓	✓	✓	✓	✓	✓	✓
[29]	✓	✓								
[15]	✓	✓								
[11]	✓	✓	✓	✓	✓	✓	✓	✓	✓	✓
[19]	✓	✓	✓	✓	✓	✓	✓	✓	✓	✓
[27]	✓	✓			✓	✓	✓	✓	✓	✓
[18]	✓	✓								
[13]– FTZs definition	✓	✓	✓	✓	✓	✓	✓	✓	✓	✓
[13] - EPZs definition	✓	✓	✓	✓	✓	✓	✓	✓	✓	✓
[13] - Free Zone definition	✓	✓			✓	✓	✓	✓	✓	✓
[46]	✓	✓								
Revised Kyoto Convention via [30].	✓	✓								
[47]	✓	✓	✓	✓	✓	✓	✓	✓	✓	✓
[48]	✓	✓								
[33]	✓	✓								
[32]	✓	✓								
[1]	✓	✓								
[31]	✓	✓								
[38]	✓	✓	✓			✓	✓	✓		
[37]	✓	✓	✓			✓	✓	✓		
[36]	✓	✓	✓			✓	✓	✓		

#### 4.2.1 Free trade zones

Free Trade Zones *(*FTZs) are the first component of *Free Zones.* It is considered the most common type of *Free Zones* [32]. FTZs are vital to facilitating trade-in globalization [32] and developing business activities [49]. Exploring factors to develop such zones plays a crucial role in the economy's prosperity [[44], [50], [51]]. Free Trade Zones are typically located within or near major seaports, airports, border areas, or along major transportation axes (marine, railways, roads) and development corridors [1,32]. Besides, most free trade zones flourish in developing countries [36].

A Free Trade Zone - or so-called ‘foreign trade zone'- is one of the world's largest economic entities that aim to facilitate importing foreign goods - that are not prohibited by local laws - by eliminating normal customs restrictions [37]. FTZ includes commercial activities, such as storage, display, processing, manufacturing, mixing, or use [52,53]. According to Hsu et al. [38], free trade zones can be described as specific areas that provide companies with some economic benefits such as tax exemption; most of the activities in FTZs are focused on industrial operations such as storage and processing. Referring to Chiu et al. [36], free trade zones can be described as special zones that aim to attract businesses and foreign investments by offering privileges such as reducing bureaucratic restrictions and eliminating normal customs tariffs and quotas. FTZs activities focus on import and export operations [36]. Free Trade Zones are considered hubs for global trade since they include many activities. According to Miyagiwa [44], they include processing, packaging, re-packaging, repairing, assembling, disassembling, sorting, distributing, storing, labeling, exhibiting, testing, etc. According to the World Bank [13], free trade zones are “*small, fenced-in, duty-free areas, offering warehousing, storage, and distribution facilities for trade, trans-shipment, and re-export operations, located in most ports of entry around the world*”*.*

Focusing on trade-related activities, Tansuhaj & Jackson [43] defined Free Trade Zones as *“a specific economic area in which an international enterprise can devote to the assignments of warehousing, packaging, the inspection, labeling, exhibition, assembly, fabrication, or trans-shipment”*. In a more comprehensive definition, Meng [15] defined FTZ as *“geographically defined in an area or zone inside a country or in a cross-border area between several countries where certain economic activities are specially allowed and where free trade and other preferential policies and privileges different from those in the rest of the country are* granted*”*. FTZ is an “*economic zone that in internarial business can utilize to optimize its supply network, it provides the advantage of operational efficiency as well as financial benefits”* [46]. In a recent definition by Organization for Economic Co-operation and Development and European Union Intellectual Property Office (OECD/EUIPO), FTZs are defined as *“designated areas that in most cases lie outside the customs jurisdiction of the economies concerned and are not subject to customs duties or most of the other customs procedures that would otherwise apply to imported merchandise*” [48].

#### 4.2.2 Export processing zones

Export Processing Zones (EPZs) are the second type of *Free Zones*. It is a recent manifestation of an organization that has existed for a long time in the form of a ‘free passages’ area. EPZs have grown dramatically since their inception in the 1950s [18,29]. In the 1980s, there was a trend toward export-oriented industrial development in many countries, particularly in Asia, as it offers firms free trade conditions and a liberal regulatory environment [13]. The original form of EPZs refers to a “*geographically restricted, and often fenced-in, enclave”* [18]. Export processing zones mainly focused on manufacturing activities in addition to other services. According to Akhtar [19], EPZs include all manufacturing activities such as electronics and apparel and other services that can be provided through a digital network like digital data processing and financial services.

Usually, EPZs are more widespread in developing countries [18,19,54]. Kusago & Tzannatos [55] listed more than nineteen concepts of the Exporting Processing Zones. The use of EPZ in different terms depends on the country, such as Free Trade Zone, duty-free zone, offshore zones [19], special economic zone, industrial Free Trade Zone, zone Franche, maquiladoras [29], free ports, and industrial districts [56]. EPZs are zones that are primarily focused on foreign markets. Such zones seek to encourage manufacturing and related activities by providing incentives and facilities aimed mainly at export markets [13]. It has two types of forms: (1) traditional EPZ: in this form, the whole area inside the zone is *“exclusively for export-oriented factories licensed under an EPZ regime”*; (2) Hybrid Export Processing Zones: these EPZs are divided into two main zones. The first is a general area open to all industries. The second is a separate EPZ area specialized only for export-oriented, EPZ-registered enterprises [13]. The EPZ generally includes a full exemption from related duties and taxes, free repatriation of profits, fewer operational barriers, more investment incentives, providing public services, exports-imports duty-free, and others [18,[56], [57],]. Such privileges are not often available for firms working under the same local customs outside the zone. The EPZ completely differs from similar concepts, such as industrial development or high-tech parks. Industrial and high-tech parks intend to develop *“growth synergies through clustering”* [58]. They usually have fewer privileges, are open to any type of activity, and do not provide complete duty exemptions [18]. According to the World Bank [13], export-processing zones are *“industrial estates aimed primarily at foreign markets”*. Besides, Hybrid EPZ includes two types of zones: (1) open to all industries and (2) separate zone dedicated to export-oriented companies [13]. Papadopoulos & Malhotra [18] defined EPZs as *“geographically defined areas within developing countries, intended to attract export-oriented foreign direct investment (FDI) by offering barrier-free environments and special incentives to firms that operate in them”.* In addition, they summarize the definitions of EPZs proposed by the United Nations Centre on Transnational Corporations (UNCTC) in 1988 and the World Bank in 1992 [39] as *“geographically defined places within a country offering a free trade environment, a liberal regulatory regime, and/or tax and other incentives oriented to attracting foreign investment and with an expectation that firms operating within them focus on export-oriented manufacturing*”.

Generally, EPZs are defined as “*geographically or juridically bounded areas in which [different levels of] free trade, including duty-free import of intermediate goods, is permitted provided that all [or most] goods produced within the zone are exported”* [45]. Referring to the International Confederation of Free Trade Unions in 1996, EPZ could be defined as *“a clearly demarcated industrial zone which constitutes a free trade enclave outside a country's normal customs and trading system benefit from certain tax and financial incentives”* [11]. In its definition for EPZs, the World Bank [39] defined the Export Processing Zones as “*fenced-in industrial estates specializing in manufacturing for export and offering their resident firms free-trade conditions and a liberal regulatory environment*”. In addition, in its analysis, the World Bank relied on the premise that *“an export processing zone is an industrial estate, usually a fenced-in area of 10 to 300 ha that specializes in manufacturing for export”*. As reported by the International Labor Organization (ILO) in 1998, EPZs are *“industrial zones with special incentives set up to attract foreign investors, in which imported materials undergo some degree of processing before being (re-) exported again”* [11]. Akhtar [19] indicated that EPZ includes a variety of activities such as bonded warehousing, assembling, export processing, trade through borders or sea, and financial services. The main difference between EPZs and FTZs is the export amount of their production. EPZs usually export between 80% and 100% [1]. If the EPZs sent their productions to the local market, authorities make policies for competition distortion purposes. The EPZ type of *Free Zones* is considered very useful [1]. It could attract many investors to the industrial *Free Zone* and its services. Some of the top EPZ examples are Shannon (Ireland, since 1959); Manaus (Brazil, since 1967); Masan (South Korea, since 1970); Bayan Lepas (Malaysia, since 1972); Batam (Indonesia, since 1978).

Based on the output of Table 2, *Free Zone* *components* can be categorized into three main groups. First, regulatory framework: This component includes regulations, rules, and procedures in addition to services that govern the operations of businesses with *Free Zones*. Second, incentives, including tax incentives, financial incentives, and privileges offered to businesses operating within *Free Zones* to encourage investment and growth. Third, infrastructure includes three components. (1) Basic infrastructure, including transport, utility, and social infrastructure. (2) Trade facilities, such as distribution centers, warehouses, and transshipment facilities. (3) Industrial facilities include packaging, assembling, manufacturing, repairing, and labeling. The infrastructure should be designed to support business operations within *Free Zones* effectively.

### 4.3 Free Zone Components

**Fig. 6:**
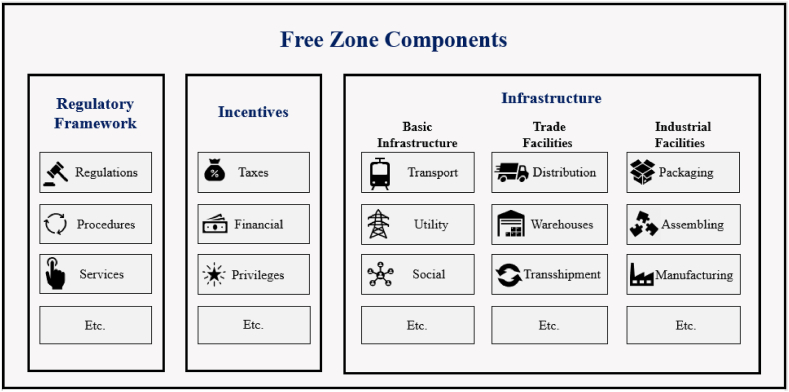
Free zone components.

*Free Zones* are designated areas within a country where economic activities, such as production and trade, are allowed to occur with reduced regulations, taxes, and duties in addition to other incentives compared to other areas within the country. *Free Zones* aim to promote economic growth and development by attracting foreign investment and businesses, providing a favorable environment for trade and investment activities. A clear concept of *Free Zones* provides policymakers and other stakeholders with a comprehensive understanding of the nature of *Free Zones* and their activity feasibility, helping design competitive incentivization programs to attract investments to be engaged in economic activities. This in turn, creates a favorable environment for businesses to invest in and expand, thereby contributing to the economic growth and development of host countries. In addition, it is crucial to develop the concept and definition to a resolution that includes the constituting components of *Free* *Zones,* which would then support the subsequent development of a methodology to assess its performance, especially with regard to sustainability performance. Accordingly, a comprehensive understanding of the components of *Free Zones* is crucial to their success. In this regard, the study aggregates the components of *Free Zones*, including the regulatory framework, incentives, and infrastructure - as shown in Fig. 6. In addition, it discusses the interaction between *Free Zone* components and draws related suggestions*.*

First, regulatory framework. As noted in Fig. 6, *Free Zones* include the following main components: regulatory framework, incentives, and infrastructure. The regulatory framework plays a critical role in the success of *Free Zones* [1,[11], [12], [13],^15^,^18^,^19^,[27], [28], [29], [30], [31], [32], [33], [47],[36], [37], [38], [39],^44^,[43], [46], [48],^45^], as noted in the *Free Zone* component Table 2. The purpose of the regulatory framework is to govern the activities within *Free Zones*, ensuring that businesses can operate smoothly and efficiently. The regulatory framework includes regulations, procedures, and services specific to the *Free Zone*, which are designed to be more favorable to businesses than those in the rest of the country. It sets out the rules and procedures that manage activities, providing users with clarity and a high level of certainty. Besides, providing services, such as customs clearance, is essential for smooth operation. A well-designed regulatory framework can attract businesses and enhance the competitiveness of *Free Zones* [1]. The strength of the regulatory framework in *Free Zones* includes its ability to provide businesses with high certainty and clarity, making it easier for them to operate. In addition, the framework offers investments with access to specialized services, such as trade and investment promotions designed to support more efficiently and effectively, leading to increased economic activity and growth. However, the regulations in *Free Zones* have weaknesses as well. For example, it may create some perception of favoritism, as businesses within the zone are given preferential treatment compared to those in the rest of the country. In addition, the framework may not always be flexible enough to adapt to changing market conditions, which could limit its effectiveness. Hence, the regulatory framework plays a vital role in contributing to the success of *Free Zones*. However, the framework should be efficient, effective, and flexible in addition to its ability to balance the needs of businesses with the broader national goals of enhancing economic growth and development.

Second, incentives. Incentives are a vital component of *Free Zones* [[59], [60], [61],^62^]. Incentives play a crucial role in attracting businesses to *Free Zones* and promoting economic activity within the zones. The purpose of incentives is to make a more attractive environment for investing by providing financial incentives, tax incentives, and other privileges. Providing such incentives can be the deciding factor for companies when choosing the best *Free Zones* for their business. Incentives vary greatly, ranging from tax exemptions to subsidies and other financial benefits. The strengths of incentives in *Free Zones* include their ability to lower the cost of doing business, making it more attractive for companies to invest in the zone. In addition, incentives can help to spur economic activity by encouraging investments, creating jobs, and increasing exports. Besides, incentives can lead to a more favorable business environment, making it easier for companies to operate and grow. However, incentives in *Free Zones* have some weaknesses. For instance, they might create some perception of favoritism, as businesses within the zone are given preferential treatment compared to those in the rest of the country. Additionally, incentives may not always be sustainable in the long term, as they may not be able to attract sufficient investment to offset the costs of providing them. Hence, incentives play a crucial role in contributing to the success of *Free Zones* by attracting businesses and promoting economic activity. However, it is essential to ensure that the incentives are efficient, effective, and sustainable in addition to their ability to balance the needs of businesses with the broader goals of promoting economic growth and development.

Third, infrastructure. Infrastructure is another key component of a *Free Zone* [1,63]. It plays a vital role in facilitating economic activity and supporting the growth and development of businesses operating within the zones. The infrastructure of *Free Zones* includes three main parts: basic infrastructure, trade facilities, and industrial facilities. They should be designed to smooth the operation of businesses and meet the diverse needs of companies and investments. In addition, the availability of trade and industrial facilities, such as distribution centers, warehouses, and manufacturing facilities, can significantly enhance the competitiveness of a *Free Zone*. By providing these components, *Free Zones* can offer investments a sufficient package, including access to the necessary resources to engage in economic activities. Basic infrastructure [1,63], such as transport infrastructure, including roads, ports, airports, and other modes of transportation, helps to facilitate the movement of goods and people in and out of the zone, while the utility infrastructure, such as water, electricity, and telecommunications services, provides access to essential services at a lower cost than in other zones. Social infrastructure, including housing, healthcare, education, and recreational facilities for employees and their families, helps to create a supportive and attractive environment for businesses [64]. Trade facilities, such as distribution, warehouses, and transshipment facilities, are designed to support the movement of goods within the *Free Zone* and beyond [[11], [12], [13],^18^,^19^,^27^,^47^,[36], [37], [38], [39],^44^,^43^,^45^], as shown in Table 2. Industrial facilities, including packaging, assembling, and manufacturing, provide businesses with the necessary infrastructure and support for manufacturing and processing products within the zone [[11], [12], [13],^18^,^19^,^27^,^47^,[36], [37], [38], [39],^44^,^43^,^45^], as illustrated in Table 2. The strengths of infrastructure in *Free Zones* include its ability to improve the business environment, making it easier and more attractive for companies to invest in and conduct trade in the zone. Besides, the infrastructure can support the growth of businesses by providing access to essential services, such as transportation and utilities, at a lower cost than in other areas. Additionally, the infrastructure can help to spur economic activity by creating jobs and attracting investment. However, infrastructure development and maintenance in *Free Zones* have some weaknesses. For example, the cost of developing and maintaining infrastructure can be substantial and might not always be sustainable in the long term. In addition, the infrastructure may not always be flexible enough to adapt to changing market conditions, with could limit its effectiveness. Moreover, sustainability should be considered when designing *Free Zone* infrastructure to reduce environmental impacts and promote long-term economic and social growth through incorporating green technologies, promoting resource efficiency, and encouraging sustainable practices among businesses.

The success of *Free Zones* might depend heavily on the interaction and coordination between its diverse components. The regulatory framework provides a clear and consistent set of rules and regulations that govern economic activities within the zone. At the same time, the incentives offer tax and financial benefits to attract investment and businesses to *Free Zones*. Furthermore, the infrastructure provides companies with the necessary physical and utility infrastructure to operate effectively. The interaction between these components is crucial to achieving the overall success of *Free Zones*. *Free Zones* should guarantee balanced integration between all components. The effectiveness of a *Free Zone* with a solid regulatory framework and attractive incentives can be reduced if the infrastructure is insufficient or inadequate, and vice versa. For example, the lack of adequate transportation infrastructure can increase the cost of doing business and limit the movement of goods in and out of the zone. In contrast, inadequate social infrastructure can impact the quality of life for employees and their families, leading to high turnover and reduced competitiveness. Regarding the overall performance, the success of *Free Zones* can be measured by various indicators such as the level of investment and job creation, the level of trade and economic activity, and the level of satisfaction among businesses and other stakeholders. When these components work together effectively, *Free Zones* can provide significant economic benefits, including increased trade and investment, job creation, and the development of new markets and industries. However, the performance of *Free Zones* can also be impacted by external factors such as economic conditions, competition from other zones, and changes in government policies. It is essential to continuously evaluate and develop the components of *Free Zones* to ensure sustainable success in meeting the needs of businesses and other stakeholders.

Finally, the study provides some suggestions related to *Free Zone* components. First, improving the regulatory framework. The regulatory framework of *Free Zones* should be regularly reviewed and updated to ensure continued relevance and effectiveness. In addition, the framework should be streamlined and simplified to reduce the administrative burden on businesses and ensure adequate protection for workers and the environment. Second, enhancing incentives. The incentives offered by *Free Zones* should be reviewed and updated to ensure continued attractiveness to businesses and investors. Besides, they should be carefully balanced to ensure that they do not have unintended consequences, such as reducing the competitiveness of other regions or sectors. Third, upgrading infrastructure. The infrastructure of *Free Zones* should be regularly reevaluated and upgraded to ensure meeting the evolving needs of businesses and other stakeholders. The focus should be on ensuring that infrastructure should be reliable, efficient, and cost-effective and addressing the needs of workers and their families. Fourth, improving interaction. The interaction between components of *Free Zones* should be reviewed and enhanced to ensure they work together effectively. Such a procedure can involve increasing collaboration and coordination between various government agencies responsible for managing *Free Zones* and improving the flow of information between businesses and other stakeholders.

## 5.0 Conclusion

*Free Zones* play an important role in promoting economic development and providing investment opportunities, where the success of *Free Zones* and their stakeholders depends on the continued evaluation and improvement of the components of *Free Zones* (regulatory framework, incentives, and infrastructure). As such, regular performance assessments of the *Free Zone* and its constituting components support evolving needs of businesses, stakeholders, and sustainability. This will help increase *Free Zones'* competitiveness and attract investment and economic activity, leading to greater benefits for all involved. Therefore, it is imperative that the *Free Zone* components and their interactions be continually monitored and improved to support the long-term success of *Free Zones*.

As a result of the growth of *Free Zones* in recent times, many efforts have been made to define the *Free Zone* and to elaborate on the differences with other economic zones. Due to the inconclusive nature of definitions, many academics and professionals have researched the topic. As such, in 2019, UNCTAD released its official generic classification of zones, in which the term ‘*Free Zone*’ was clearly defined. In this generic definition, the term ‘special economic zone’ was considered a comprehensive term for all types of zones, including *Free Zones*. Despite the role of the UNCTAD [1] and Bost [10] frameworks for economic zones in defining the ‘*Free Zones*’ concept, it did not provide a sufficient description of the *Free Zone* components. In this regard, this review provided a historical narrative of the definitions and terminologies related to *Free Zones* and their types, reaching up to the period of the UNCTAD official framework in 2019. It further defines and conceptualizes *Free Zones* by aggregating them into their constituting components and highlights how they interact with one another. Accordingly, a *Free Zone* can be considered as a specific geographic scope where governments support economic activities -whether commercial or industrial-through offering a series of privileges focusing mainly on exemptions from customs duties and tax incentives and extending to include a wide range of support such as facilitating administrative procedures, permits, licenses, and consultations.

The main contributions of this study can be summarized as follows. First, providing general knowledge about the terminology of *Free Zones*. Second, presenting classifications and types of economic zones and offering insights into what distinguishes *Free Zones* from other economic zones. Third, aggregating the *Free Zone* into its constituting components, including regulatory framework, incentives, and infrastructure. Finally, providing insight into future research by identifying relevant challenges and gaps.

There are some limitations to this study. One of the main challenges was the scarcity of relevant resources and the absence of a standardized term for *Free Zones* before 2019. As a result, this study could not follow a systematic approach to a literature review in the first part regarding *Free Zones* terminology. Instead, it relied on official reports from relevant organizations and other relevant literature. Second, the methodology used to propose *Free Zone* components was limited to Bost's model for *Free Zones* in 2010 [10] and the UNCTAD general framework for special economic zones in 2019 [1]. Accordingly, to identify *Free Zones*, the study was limited to including the two components: Export Processing Zones and Free Trade Zones. Third, although the study was limited to suggesting and explaining the components of *Free Zones*, it did not delve deeply into detailing them. Hence, they can be promising opportunities for future research. Accordingly, the study hopes to have succeeded in drawing future research attention toward more investigation and contributions in the field.

While UNCTAD [1] and Bost [10] have provided insight into *Free Zones*, there remains a further opportunity for understanding and defining *Free Zones,* beyond what has been highlighted in this review to provide the necessary foundation to support sustainability assessment. Furthermore, understanding the dynamics between the different components of *Free Zones*, within the context of performance and sustainability assessment would be an interesting opportunity for future research. This would be complemented by developing the necessary tools and methodologies to undertake such assessment, with regard to sustainability. Finally, applying the aforementioned objectives in a case study is important for validating the proposed sustainability assessment built on the component approach. There are many cases around the world that would provide for a good case study. For instance, the Middle East or China, which have both seen tremendous economic growth and interest in trade facilitated by *Free Zones*. Undertaking the aforementioned research endeavors would reduce ambiguity as it relates to defining *Free Zones*, enhance the relevant knowledge, align policymakers, and improve performance analysis.

## Author contribution statement

All authors listed have significantly contributed to the development and the writing of this article.

## Data availability statement

No data was used for the research described in the article.

## Declaration of interest's statement

The authors declare no competing interests.
